# Natural and Magnetic Circular Dichroism From the Infrared to the UV of a Hetero[4]helicene Radical Cation

**DOI:** 10.1002/asia.202401752

**Published:** 2025-03-12

**Authors:** Marco Fusè, Michela Lupi, Ewa Machalska, Giuseppe Mazzeo, Sergio Abbate, Julien Bloino, Caterina Viglianisi, Stefano Menichetti, Giovanna Longhi

**Affiliations:** ^1^ Università degli studi di Brescia Dipartimento di Medicina Molecolare e Traslazionale (DMMT) Viale Europa 11 25123 Brescia Italy; ^2^ Università di Firenze, Dipartimento di Chimica “Ugo Schiff” Via Della Lastruccia 13 50019 Sesto Fiorentino (FI) Italy; ^3^ Istituto Nazionale di Ottica (INO), CNR Research Unit of Brescia, c/o CSMT VIA Branze 45 25123 Brescia Italy; ^4^ Scuola Normale Superiore Piazza dei Cavalieri 7 56126 Pisa Italy

**Keywords:** hetero[4]helicenes, VCD, NIR-CD, MCD, computational chiroptical spectroscopy

## Abstract

Dithiabridged triarylamine hetero[4]helicenes are an interesting class of chiral compounds characterized by a high racemization barrier which makes them an attractive material for several applications. Their rich redox chemistry allows one to obtain radical cations stable at room temperature, that can be isolated in two configurationally stable enantiomers. While several studies have been reported on systems with transition metals possessing low‐lying electronic states, the characterization of purely organic open‐shell systems by VCD is still scarce. This may be due to the instability of radical‐cation species and to the dark color assumed by the solution at the high concentration required by VCD. The oxidation of this molecular system shifts the first electronic transition towards the Near‐IR (NIR) region, at approximately 1250 nm. By combining ECD, VCD, MCD, NIR‐CD and computational methodologies rooted in density functional theory, we have investigated one molecule of this class of compounds and shed some light on the chiroptical properties accessible via redox‐switch.

## Graphical abstract

By combining ECD, VCD, MCD, NIR‐CD and computational methodologies, we investigated the changes on the chiroptical properties accessible via redox‐switch on a hetero[4]helicenes molecule and its radical cation.

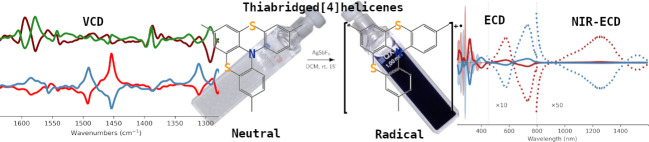

## Introduction

Helicenes are a class of compounds that has been extensively investigated for their intrinsic chirality and their photophysical properties, finding applications in several fields such as material science,[^1^, ^2^, ^3^, ^4^, ^5^, ^6^] asymmetric synthesis,[^7^, ^8^, ^9^] medicinal chemistry,[^10^, ^11^, ^12^] and molecular recognition.^[13]^ Within the broad family of helicenes, dithia‐aza[4]helicenes (DTA[4]H) are an interesting class of compounds that have attracted considerable interest in the last years. DTA[4]H can be described as two phenothiazines sharing an aryl ring and a nitrogen atom. The long carbon–sulfur bonds distort the structure, thus forcing the other aryl rings out of the common plane and defining a helical pitch. DTA[4]Hs are characterized by an interconversion barrier higher than that of [5]‐helicenes and approaching the one of [6]‐helicenes, making the four ring members of the class configurationally stable at room temperature.^[14]^ More precisely, in Ref. [14] the energy barriers of racemization $\Delta G^{‡}$ =131.9–132.6±0.5 kJ/mol^−1^ were obtained in decalin in the 121–145 °C temperature range (see details in reference), permitting to conclude that helicity reversal barrier in the thia‐bridged hetero[4]helicene is between that of the parent [5]‐helicene ($\Delta G^{‡}$ =101 kJ/mol^−1^ at 20 °C) and [6]‐helicene ($\Delta G^{‡}$ =151 kJ/mol^−1^ at 27 °C). Their exceptional stability allows their enantiomeric resolution by HPLC on a chiral stationary phase and their storage in enantiopure form.

DTA[4]Hs were initially designed starting from the triarylamines, and, like their parent compound, are characterized by remarkable photophysical properties. The chiroptical properties as emitter of dithia‐helicenes from four up to six rings were recently investigated by recording also the circularly polarized luminescence (CPL) for several molecules.[^15^, ^16^]

Another unique feature of this class of helicenes is their ability to be reversibly oxidized to the corresponding radical cations, which are stable at room temperature also in solution.[^17^, ^18^] Considering the ease of generating the radical cations and their stability as paramagnetic chiral molecules, they have been recently used to develop chiral molecule‐based spintronic devices, exploiting the very popular chirality induced spin selectivity (CISS) effect.[^19^, ^20^]

Due to the intrinsic reactivity of radical species, vibrational circular dichroism (VCD) spectra of such species are not common in the literature.[^21^, ^22^] Open‐shell systems were investigated by VCD in particular when a transition metal was present in the molecule. In this latter case, the transition centered on the metal (i. e., d–d) can interact with the vibrational states giving rise to enhanced VCD signals.[^23^, ^24^, ^25^, ^26^] Purely organic open‐shell systems are less investigated by VCD; in some cases they have been produced directly in the spectroscopic cell by electrochemical oxidation or photochemical reaction of the neutral species.[^27^, ^28^, ^29^] Nonetheless, we would like to remind that metal‐complexes bearing helicene systems had already been studied with VCD and ROA.[^30^, ^31^, ^32^] Besides, other numerous important papers on chiroptical properties on organometallic helicene systems exist.[^33^, ^34^]

In this paper we re‐investigate from a chiroptical perspective one of the simplest member of the DTA[4]H family, which presents C_2_ symmetry. We will focus on the effect of oxidation on the chiroptical properties in a system where only minor geometrical changes are observed. In this process we will extend the available spectroscopic data in spectral range (near‐infrared) and techniques (MCD and VCD).

The paper is organized as follows: in the first part the electronic transitions of the neutral and radical‐cation species are investigated by ECD and MCD (electronic and magnetic CD, respectively) from the NIR to the UV region. In the second part, the influence of the electronic changes occurring upon oxidation on the vibrational absorption (VA) and VCD spectra are investigated. In the last section we discuss on the band shape observed in the ECD spectra in the NIR spectroscopic region.

## Results and Discussion

**Scheme 1:**
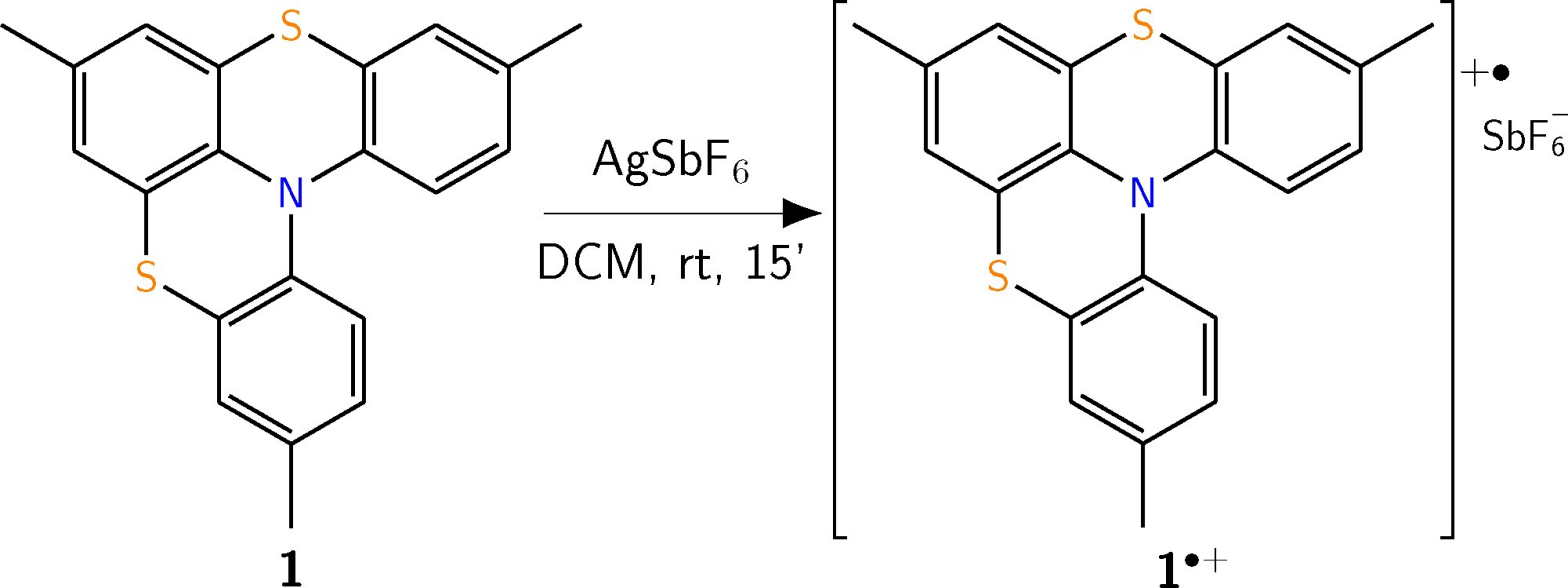
Reaction scheme to obtain **1**⋅^+^.

The two enantiomers of the neutral molecule were separated by chiral‐chromatography as previously reported.[^14^, ^35^] As shown in Scheme 1, the oxidation of the parent compound **1** to the radical‐cation **1**⋅^+^ is obtained by adding AgSbF_6_ in CH_2_Cl_2_ at room temperature. In ca. 15 minutes **1**⋅^+^ is obtained and the configuration of the neutral form is retained and remains stable.^[14]^

As shown in Table 1, and in agreement with X‐ray data reported in Ref. [36], the oxidation of **1** leads to a reduction of the helical pitch (compare for instance the C_17_C_5_C_9_C_27_ torsion angle and C_19_‐C_25_ interatomic distance values between the two species, reported in Table 1), leaving “flat” the nitrogen atom where the spin‐density is localized in the radical‐cation form (see Figure S3b in Supporting Information).^[17]^ Instead, the two sulfur atoms deviate slightly more from the mean plane defined by the rear phenyl ring in the radical‐cation form. Overall, the observed deformation between the two oxidation states is not dramatic, allowing a direct comparison between the two structures.

**Table 1:** Comparison of experimental geometrical parameters (Å and degrees) of **1** and **1**⋅^+^ (Scheme 1) with calculated parameters from the structures optimized at the B3PW91/TZVP level of theory. The atom numbering is reported in Figure S3a, while graphical representations are given in Figures S3c and S3d of the Supporting Information.

	Neutral		Radical	
	Calc.	Exp.^[a]^	Calc.	Exp.^[b]^
N_1_‐C_4_	1.409	1.420	1.395	1.411
N_1_‐C_14_	1.410	1.425	1.409	1.413
S_2_‐C_5_	1.779	1.760	1.759	1.755
S_2_‐C_15_	1.779	1.758	1.755	1.741
C_19_‐C_25_	3.205	3.147	2.987	2.918
C_5_S_2_C_15_	98.7	99.1	99.6	100.1
S_2_C_5_C_9_S_3_	−11.7	−9.8	−18.0	−17.6
C_17_C_5_C_9_C_27_	58.7	55.3	45.9	41.4

[a] Experimental data from Ref. [14] and [b] from Ref. [17].

### ECD and MCD Spectra

**Figure 1:**
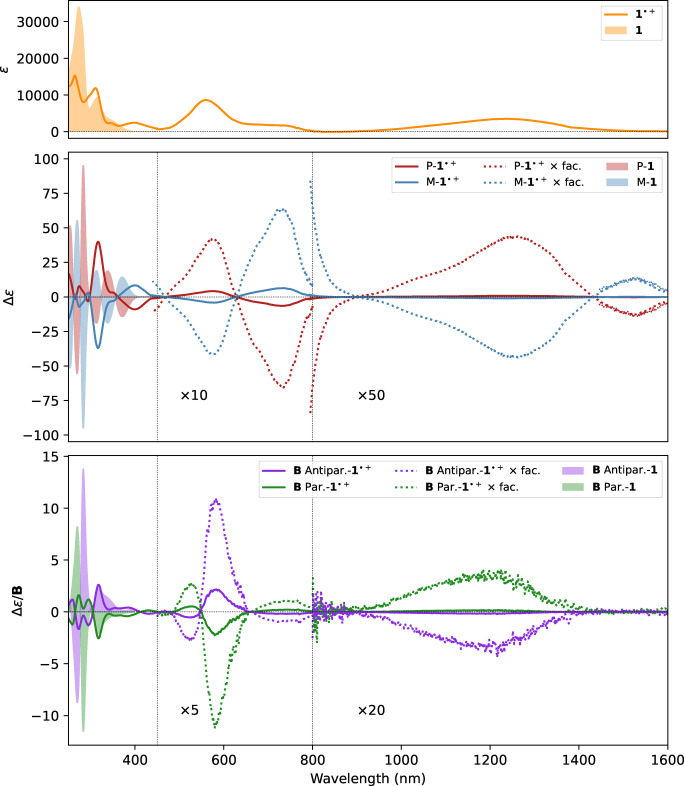
UV‐Vis (top panel), ECD (middle panel) and MCD (bottom panel) experimental spectra of **1** and **1**⋅^+^. Measurements were carried out on a CH_2_Cl_2_ solution in the 250–800 nm range and CD_2_Cl_2_ in the 800–1600 nm one. **1**’s spectra are reported as shaded areas and **1**⋅^+^’s spectra in lines. MCD spectra were recorded on racemic samples. Dotted lines represent **1**⋅^+^ spectra multiplied by intensity factors reported within the panels. The boundaries between the different scaling factors are represented as thin vertical lines. *ϵ* and Δ*ϵ* are given in 10^3^ mol^−1^ cm^2^ units and in the bottom panel B is expressed in Tesla (see Experimental Section).

The chiroptical properties of the neutral compound were previously investigated by ECD, VCD and CPL,[^14^, ^15^] while those of a related radical‐cation derivative were investigated by ECD and optical rotation by Gliemann et al.^[36]^ Our molecular system is closely related to that one, the only differences being the methyl groups instead of the *tert*‐butyl groups of Ref. [36]. The UV‐Vis and the ECD spectra reported in Figure 1 are very similar to the ones reported in Ref. [36]. Indeed, both the methyl and *tert*‐butyl groups have little influence on the low‐energy transitions which are centered on the hetero‐helicene scaffold.

Upon oxidation, three new absorption bands appear in the spectrum above 450 nm up to the NIR region. The spectra were recorded up to 2000 nm to exclude the presence of other transitions at lower energies. The ECD spectra of the two enantiomers of the radical‐cation **1**⋅^+^ exhibit mirror image shapes in all the recorded spectral ranges. Surprisingly and differently from already published spectra on the similar compound mentioned above, the ECD spectrum in the longest wavelength portion of the NIR region shows two different bands, which, for the *P* configuration, are negative (1540 nm) and positive (1250 nm), the second one being three times more intense than the first one. Data were confirmed by two independent sets of measurements taken with two different InGaAs detectors operating on overlapping ranges (see Experimental Section for details). Despite the quite intense observed absorption peaks, the CD signal recorded in the NIR region is quite low in intensity. The experimental dissymmetry factors in correspondence of the first three observed absorption bands are 2.5 ⋅ 10^−4^, 1 ⋅ 10^−3^ and 5 ⋅ 10^−4^, respectively. Conversely, the *g* value for the CD feature at about 1540 nm exceeds 10^−2^, mainly because absorption is almost vanishing. Compared with the ECD spectra of **1** (reported as shades in Figure 1), the ECD spectra of **1**⋅^+^ are overall less intense.

**Figure 2:**
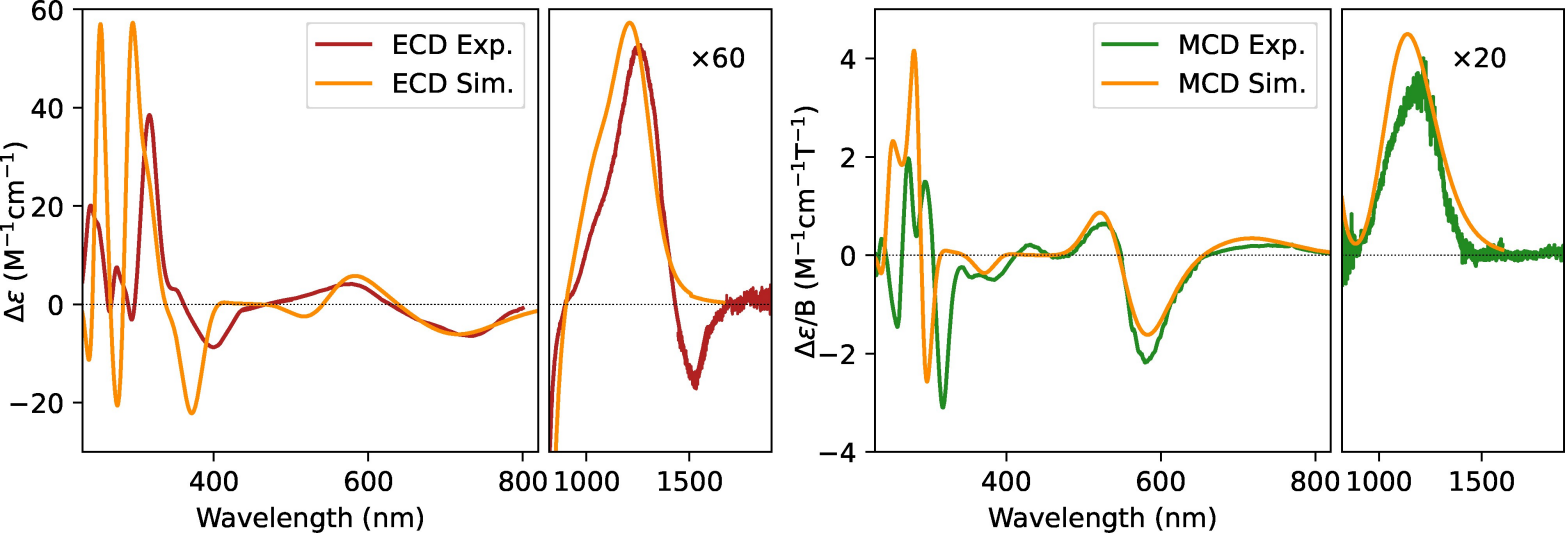
Comparison of experimental and simulated ECD and MCD spectra of **1**⋅^+^ (*P* enantiomer and racemic form, respectively). Notice the intensity factors between the two spectral regions.

The spectra were simulated at the TD‐DFT level with the combination of the B3PW91 functional and the TZVP basis set. The **1**⋅^+^ results are reported in Figure 2 and are in excellent agreement with the experimental ones (simulated spectra of the neutral specie are reported in Figure S4 in Supporting Information). The first four transitions responsible for the first three bands of **1**⋅^+^ observed in the absorption spectrum, are all associated with transitions towards the SOMO (single occupied molecular orbital), which originate from the three phenyl rings. In the neutral form the three transitions between 280 and 400 nm are associated to transfer from the nitrogen to the rings. Both in the radical cation and in the neutral form, the more energetic transitions involve the sulfur atoms and are the ones where the intensity of the ECD signals is more reduced with respect to the neutral form. Interestingly, the simulated ECD spectra in the 900–1600 nm region is not predicting any transition in correspondence of the first weak band at about 1540 nm in the experimental spectra. In order to improve the accuracy of the simulation, the first transition was also simulated including vibronic effect within the vertical hessian approach. The dependence of dipole transition moments on the nuclear coordinates was also accounted for, including the so‐called Herzberg–Teller terms (AH|FCHT). The vibronic progression sensibly improves the band shape, nonetheless the weak CD band at 1540 nm is not reproduced yet (see Figure S5 in Supporting Information for a comparison between pure electronic and vibronic simulation).

The analysis of TD‐DFT results and of the computed dipole transition moments allowed the classification of the transition according to the A‐ and B‐species. The results are summarized in Table S1 in Supporting Information, while here we just want to highlight the B symmetry of the transition at 1200 nm (see the last paragraph).

Magnetic circular dichroism (MCD) is a well‐established technique to investigate the electronic and molecular structure gaining increasing attention.^[37]^ Both achiral and chiral molecules preferentially absorb right‐ or left‐circularly polarized light in the presence of a static magnetic field directed along to the propagation of the light beam. The sign of the signal is reverted when the magnetic field is inverted. Presenting both positive and negative peaks, MCD spectra are more resolved than the absorption ones, and for chiral samples they can provide complementary or supplementary information to ECD spectra. MCD has proven to be sensitive to supramolecular arrangement, and it was recently employed to analyze the aggregation state.^[38]^ MCD spectra have been recorded in the ultraviolet, visible and NIR regions.^[39]^ However, in the NIR region, MCD are generally recorded on samples of biological interest employing strong magnetic fields.[^37^, ^40^, ^41^, ^42^, ^43^]

In order to investigate further the electronic transitions of the system in its neutral and radical‐cation forms, we measured the MCD spectra on a racemic sample in the range 250–1600 nm. The spectra obtained with parallel and antiparallel orientations of the magnetic field are reported in the bottom panel of Figure 1. A magnification of the 250–450 nm region is provided in Figure S1 of the Supplementary Material. In the UV region, the MCD spectrum of the **1** presents many features at high energy: *e. g*. in correspondence with the single absorption UV peak at 318 nm MCD highlights the presence of two transitions. The same is observed for the radical‐cation form, which shows intense MCD transitions in the visible range, where the intense peak at 580 nm is characterized by a doublet, revealing the presence of two transitions. On the contrary, MCD in the NIR region highlighted the presence of only one feature centered at 1250 nm. This MCD band matches the absorption band at 1250 nm and the high‐energy component of the first ECD doublet.

To confirm the assignment of the observed MCD bands as result of monomeric species in solution, the MCD spectra were simulated at the TD‐DFT level. Considering the absence of degenerate states by symmetry, which implies that only “𝔅 terms” give contribution,^[37]^ we decided to employ the sum‐over‐states (SOS) approach as developed by Štĕpánek and Bouř,[^44^, ^45^] which has proven very efficient and produces accurate MCD spectra from electronic excited stated obtained at the TD‐DFT level of theory.[^46^, ^47^]

Results for **1**⋅^+^ are reported in Figure 2, while the simulated spectrum of **1** is reported in Figure S4 in Supplementary Material. The simulated spectra are in excellent agreement with the experimental ones. In particular the agreement between the experimental and simulated MCD spectra in the NIR region is remarkable.

### VCD and VA Spectra

**Figure 3:**
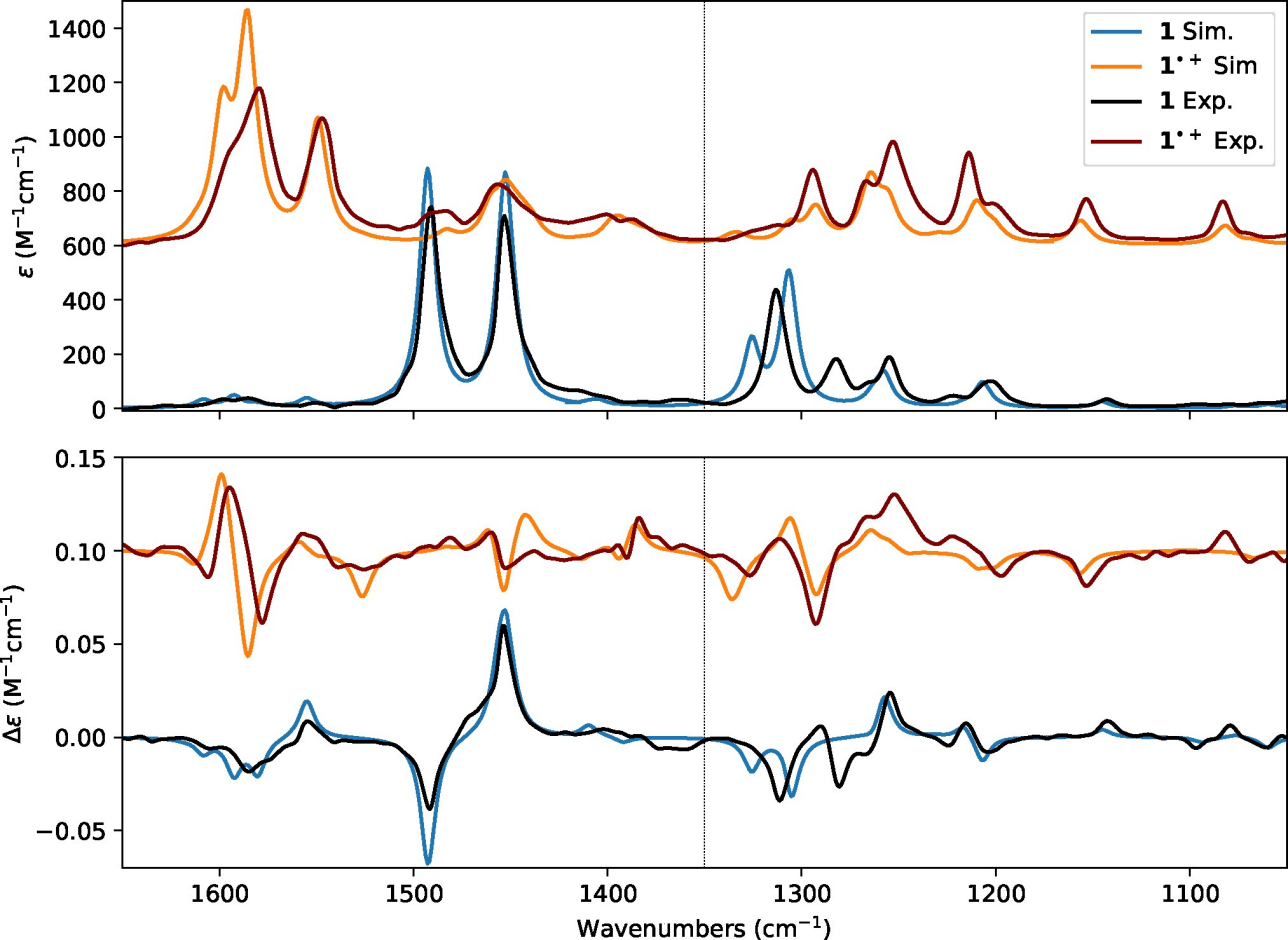
Comparison of experimental and simulated absorption IR (top panel) and VCD spectra (bottom panel) of **1** and **1**⋅^+^ *P* enantiomer. The calculations were performed at the B3PW91/TZVP level of theory within the harmonic approximation. The spectral band‐shape was obtained by applying Lorentzian distribution functions with 5 cm^−1^ half‐width at half‐maximum. Scaling factors of 0.985 and 0.97 were applied to the harmonic frequencies below and above 1350 cm^−1^, respectively.

As noted in the introduction, VCD spectra of radicals (either in neutral, anionic or cationic form) are scarce in the literature. In our case, thanks to the stability of the radical cation, we were able to measure the VCD spectra of the radical cation **1**⋅^+^ in CD_2_Cl_2_ solution. The IR absorption and VCD spectra of **1** and **1**⋅^+^ in the fingerprint region are reported in Figure 3, where the half‐difference of the VCD spectra are shown ($\frac{1}{2}\left[\Delta \epsilon \left(P\right)-\Delta \epsilon \left(M\right)\right]$ ), while the spectra of both enantiomers are reported in Figure S2 of the Supporting Information. Characteristic bands clearly denote the difference between the molecule in the two oxidation states. Particularly informative is the region above 1300 cm^−1^: distinct peaks are present both in the absorption and VCD spectra. While **1** exhibits two intense bands between 1400 and 1500 cm^−1^, actually the most intense signals over the spectral range, with only small signals between 1550 and 1620 cm^−1^, the situation is opposite for **1**⋅^+^. The signatures are even clearer in the VCD spectra: **1** presents an intense doublet (−/+ from high to low wavenumbers for the *P* configuration) between 1500 and 1400 cm^−1^, while **1**⋅^+^ presents a sequence of four bands (−/+/−/+ from high to low wavenumbers for the *P* configuration) between 1620 and 1450 cm^−1^.

When comparing the VCD spectra of a neutral and a radical‐cation species, it is worthwhile to recall that when an electronic transition approaches in energy the range of the vibrational transitions, interference between the two phenomena may occur. This interaction may lead to some enhancement of the intensity associated to the vibrational transitions. As already mentioned in the introduction, this was observed and in some cases exploited in particular for electric‐dipole‐forbidden and magnetic‐dipole‐allowed transitions, with a significant enhancement of the VCD signal. Nafie provided a theoretical treatment of this phenomenon within the complete adiabatic vibronic coupling formalism for fundamental harmonic transitions, extending the theoretical expression for IR and VCD beyond the magnetic field perturbation (MFP) theory by Stephens^[48]^ to those systems where low‐lying electronic states (LLESs) are present (see appendix).^[49]^

Without an explicit evaluation of the contributions, the perturbation term, which virtually requires summation over all the excited states, is weighted by

(1) $$\left[\frac{\omega_{a}^{2}}{{\left(\omega_{eg}^{0}\right)}^{2}-\omega_{a}^{2}}\right]$$

where $\hbar \omega_{eg}^{0}$ is the energy difference between the electronic ground and excited states, and $\omega_{a}$ is the frequency of the “resonant” vibrational mode. To reduce the probability of unphysical results in resonance‐like conditions, imaginary damping factors need to be added (Ref. [49]). In any case, the contributions to the perturbative term decrease rapidly as soon as the vibrational and electronic transition energy diverge. In the absence of large dipole transition moments the LLES must lie very close in energy to the vibrational region considered. Analyzing the fingerprint region of our system, as in all the other purely organic systems reported in the literature, one does not expect to see any enhancement on the signals (see also Ref. [28]).

As a matter of fact, even the CH‐stretching fundamental transitions do not appear affected by the presence of the transitions in the NIR region. The VA spectra of the two species were detected, whereas the VCD signals of the two species were so weak that it was not possible to record their VCD spectra. In Figure S8 of the Supporting Information the simulated spectra in the CH‐region are reported. One may appreciate that the VA calculated intensity of the radical cation transitions are much weaker than the already weak intensity calculated for the neutral form.

In Figure 3, the simulated spectra at the harmonic level of the two species are also reported. To match the experimental spectra, two different scaling factors, of 0.985 and 0.970, were applied to the harmonic frequencies below and above 1350 cm^−1^ (marked with a vertical dashed line in Figure 3), respectively. Within the MFP theory, the agreement between the experimental and simulated spectra is excellent. From an analysis of the DFT calculations, it was possible to assign the bands to the vibrational modes of the two species. Even though the optimized geometries do not possess an exact C_2_ symmetry, it may be useful to classify the normal modes according to the C_2_ point group.

Eight transitions are present in the region between 1400 and 1500 cm^−1^, mostly involving CH bending and CN stretching modes. In particular, the two most intense transitions are associated to the anti‐symmetric in‐plane CH bending on the two aromatic rings in the helicene (1480 cm^−1^, *B*‐species), and to a combination of in‐plane CH symmetric bending on the “rear” ring and the CN stretching mode for the transition at 1450 cm^−1^ (*A*‐species), respectively. The four VCD bands observed for **1**⋅^+^ in the region between 1450 and 1620 cm^−1^ are all associated to CC stretching vibrations. From higher to lower wavenumbers: the in‐phase C=C stretching modes on all the aromatic rings in the molecules symmetric with respect to the C_2_ axis, the out‐of‐phase C=C stretching on the two equivalent helicene aromatic rings (*B*‐species), the CC stretching of the rear ring of the *A*‐species and the in‐phase CC stretching on two equivalent helicene aromatic rings which are anti‐symmetric with respect to the C_2_ axis. A graphical representation of some relevant normal modes for the forthcoming discussion is reported in Figure S6 in the Supporting Information.

Considering the small geometrical deformation observed upon oxidation, we decided to investigate how the change from a closed‐shell to an open‐shell system can affect the normal modes and the IR absorption and VCD intensity. To compare two different sets of normal coordinates we used the transformation proposed by Duschinsky[^50^, ^51^] to express one set of mass‐weighted normal coordinates (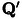 ) with respect to the other (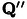 ):
(2)

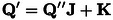




where J is the Duschinsky matrix and K the shift vector, whose definitions depend on the type of reference coordinates. In Cartesian coordinates,
(3)

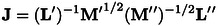



(4)

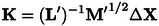




where L is the transformation matrix from mass‐weighted Cartesian to normal coordinates, M is the diagonal matrix of atomic masses, and ΔX is the difference between the equilibrium Cartesian coordinates of the two systems. Thus if 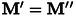 :
(5)

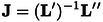




**Figure 4:**
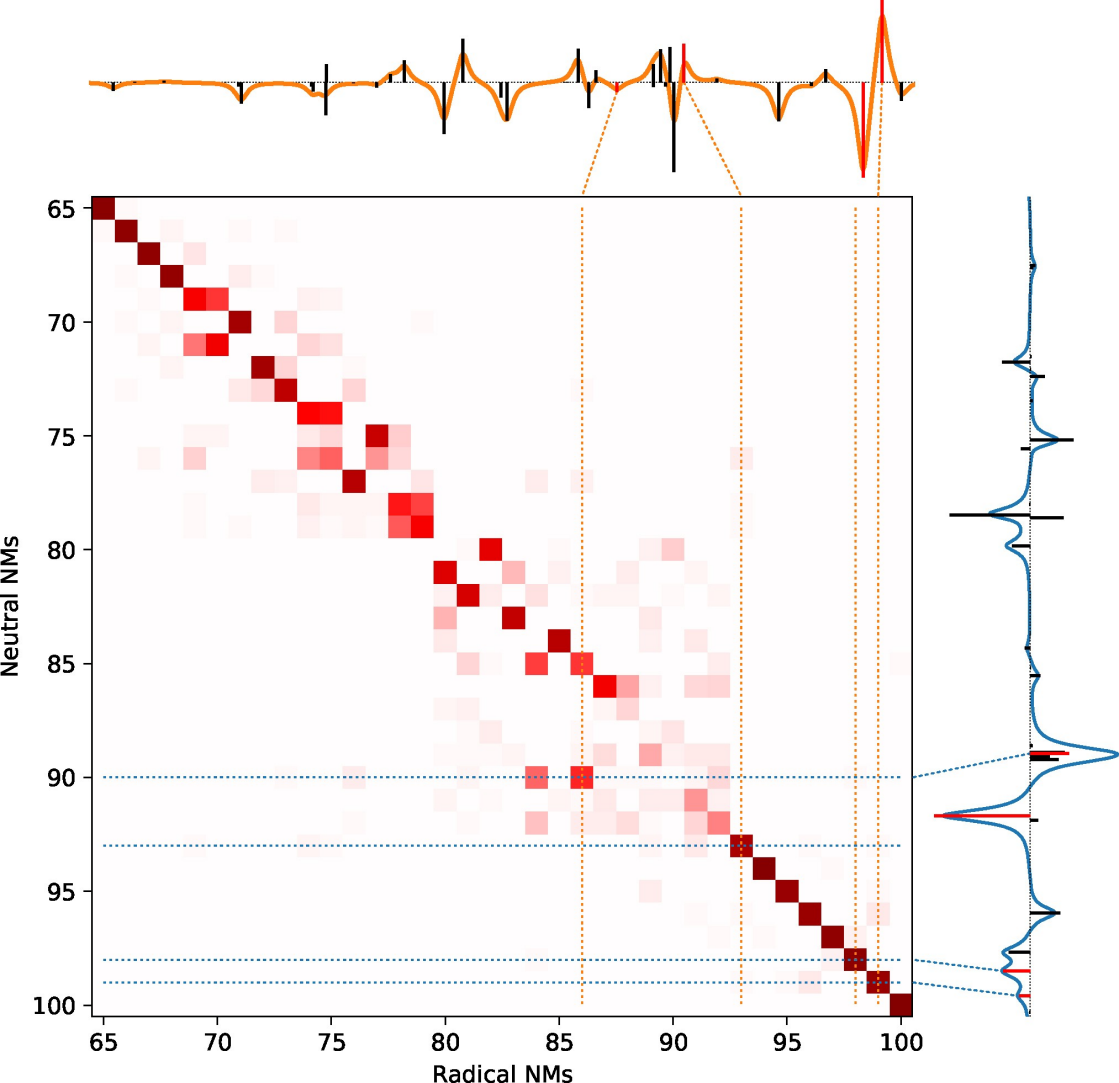
Duschinsky matrix of a selected range of normal modes to enlighten NMs correlation between **1** and **1**⋅^+^. On top, the VCD spectra of **1**⋅^+^ is reported, while on the right, the VCD of **1** is reported. Within the spectra, the most relevant transitions, which are commented in the text, are represented with red lines and their elements highlighted with dashed lines in the matrix. The squared elements of ***J**_ij_* are calculated and represented with squares of darkness proportional to the strength of the element (based on the value 0: white, 1: dark red). Hence, a mode swap is displayed as dark off‐diagonal element, while a mixing is displayed as multiple blocks of fading red.

In Figure 4, a selected range of the Duschinsky matrix between **1** and **1**⋅^+^ normal modes is reported. Among the analyzed normal modes, only one of them is strongly affected by the oxidation of the molecular system, showing large off‐diagonal components between the two systems **1** and **1**⋅^+^: NM 90 in the neutral form is a combination of NM 84 and 86 in the radical‐cation form. The other NMs are superposable between the neutral and the radical‐cation forms. In particular, the change in the molecular state shows little influence on the form of the C=C stretching normal modes (NMs 98 and 99). The same transitions are present, with little difference in energy between **1** and **1**⋅^+^, and only the changes to the electronic contributions enhance or weaker the peaks in the spectrum. In Tables S2–S6 of the Supporting Information, the energy values of the transitions are reported together with the electric and magnetic dipole transition moments. The dipole transition moments were separated in their electronic and nuclear contributions. As expected from the analysis of the Duschinsky matrix, the nuclear contributions are very similar between the two species, while the electronic contributions to the electric dipole transition moments change remarkably. On the other hand, the magnetic dipole transition moments show only little changes with respect to the electronic contributions between the two oxidation states. In all the five reported transitions, the electronic contributions are enhanced in the radical‐cation form. This leads to opposite situations in the two regions, as can be observed in the highest energy part of Figure 3. In the CH bending region (NMs 90 and 93), the two most intense signals are characterized by a strong electric dipole transition moment, which is almost canceled out in the radical‐cation form by nuclear and electronic contributions of opposite signs. The picture reverses in the CC stretching region (NMs 98–100), where the electronic contributions are enhanced in the radical‐cation form and with contributions of the same sign as the nuclear one, leading to strong signals both in absorption and VCD spectra.

### NIR‐CD Spectra

As mentioned above, the ECD spectra of **1**⋅^+^ in the NIR region shows a bisignated band shape, which is not predicted by TD‐DFT simulations when only one molecule is included. Considering the relatively high ϵ of the band at 1250 nm, and the low dissymmetry factor, this band could be sensitive to the presence of a second molecule through dipolar type interactions.

In order to investigate the possible arrangements between two charged **1**⋅^+^ molecules, we looked at the crystal structure determinations of the radical‐cation species reported in the literature.[^14^, ^17^, ^36^] Both the crystal structures of **1**⋅^+^ and the one of the *tert*‐butyl derivative of the radical cation were reported for racemic crystals. Nonetheless, even in these conditions, regular arrangements of molecules of the same helicity were observed. Between molecules of the same configuration, two main arrangements are noted: one is a columnar‐like arrangement, with a stacking of the rear phenyl ring and one of the front phenyl ring, a second one is mediated by the presence of the SbF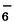 counter ion, which keeps close two molecules interacting with it through the nitrogen atom. In addition to these two arrangements, a third one is observed in the crystal of the *tert*‐butyl substituted helicene. In this last case, two molecules directly interact through the nitrogen atoms, suggesting a pairing of two opposite spin states (see Figure S9 in the Supporting Information).

Thus, we extracted these three arrangements for the **1**⋅^+^ dimer from the crystallographic structures and we simulated the NIR‐CD spectrum for the first low‐energy transitions at the TD‐DFT level of theory. In one case, the SbF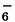 counter ion was included in the simulation to rule out any contributions thereof to the spectrum and the different spin matching between the two molecules was also considered. The results are reported in Figures S10 and S11 of the Supporting Information. Regardless of the simulation results, the first band turned out to be particularly sensitive to the interactions with a second molecule in solution, hence suggesting possible aggregation in the solution.

In this case, the band shape is expected to be dependent on the concentration of the sample and the temperature. To explore this possibility, the ECD spectra were recorded at different temperatures in the range −15 to 20 °C and ECD and NIR‐ECD spectra were recorded at different concentrations from 1.5×10^−2^ to 1.5×10^−4^ M. In the examined temperature range, the band shape did not change in the 250–850 nm region, while a little dependence of the intensity of the bisignated bands at 1250–1450 nm was observed.

Nonetheless, the limited experimental evidences as well as the simulation results call for caution in the explanation.

An alternative interpretation is that the observed bisignated band shape can be connected, for a monomer, to a Fano‐Hansen effect, which is the result of the resonant interference between a discrete transition and a continuous manifold,[^52^, ^53^] the continuous feature being the vibronic distribution of the electronic transition, and the discrete state being a high‐overtone or ‐combination (at least three quanta) vibrational localized transition. As described by Hansen, in a C_2_ molecular system the interference between the two transitions with the transition moments both polarized in the same direction with respect to the C_2_ axis can lead to a bisignated signal. This restricts the vibrational transition pool of candidates to those with a B symmetry. The resulting shape of the predicted band resembles that observed here in the experimental spectra.

## Conclusions

In this work we have investigated the chiroptical properties of a dithia‐aza[4]helicene in its neutral and radical‐cation form. The two species are characterized by a similar geometry, which allowed a direct comparison of the effect of electronic distribution differences on the chiroptical properties.

The VCD spectra of the two oxidation states were recorded in the finger print region, and specific features for each species were identified. Despite the open shell nature of the radical cation, the significant energy difference between the electronic transition at lower energy and the fundamental vibrational transitions did not lead to any perturbation of the VCD signals. The excellent agreement between the experimental and DFT simulated spectra allowed us to assign the differences mostly to the electronic contributions to the electric dipole transition moments.

The ECD and MCD spectra were recorded in the UV‐Vis and NIR regions. Upon oxidation, new bands appeared in the spectrum of the radical cation, which were assigned to transitions towards the SOMO orbital. The combination of the two techniques allowed us to better investigate the latter transitions; MCD spectra highlighted the presence of two transitions beneath the radical cation absorption intense peak at 580 nm. At lower energy, the ECD spectra of the radical cation in the NIR region showed a characteristic bisignated band shape, which was not observed previously for similar compounds. TD‐DFT simulated ECD and MCD spectra were in excellent agreement with the experimental ones, in all the spectroscopic range with the only exception of the first ECD band in the NIR region.

In order to interpret the latter observed band shape, we have considered two different mechanisms: the interaction between two radical‐cation molecules mediated by the presence of counter ions or an interference between the vibronic manifold of an electronic transition and a vibrational overtone. Further investigations are needed to clarify the origin of the observed doublet.

## Experimental and Computational Details

In the 250–800 nm range, CD and MCD measurements were conducted with a Jasco 815SE instrument apparatus, employing quartz cuvettes with 2 mm path length, on solutions from 10^−5^ to 3×10^−4^ M. MCD data were obtained with the PMCD‐586 commercial module, producing 1.5 T magnetic field. 10 scans were taken for every measurement; when needed, up to 20 scans were taken. In the NIR region, we used a home‐built dispersive instrument with a −20 °C cooled InGaAs detector, working in the range 1600–800 nm and an extended InGaAs detector cooled at −30 °C in the range 2400–1450 nm.[^54^, ^55^] The concentration was 10^−3^ M in ${\mathrm{CD}}_{2}{\mathrm{Cl}}_{2}$ solutions and the number of accumulated scans was 25 with absorption baseline (ABL) spectra subtracted from CD spectra (see Refs. [54, 55]) and solvent spectra run in the same conditions were subtracted out in the final stage. MCD spectra in the NIR were recorded with a home‐built cell holder hosting neodymium‐based magnets, which produced ≈0.6 T magnetic field in a 0.2 cm pathlength quartz cell.^[56]^ The MCD traces reported in this work are obtained by subtracting from the CD spectra recorded at **B** ≠ 0 in the two orientations the CD spectra recorded at **B**=0.

VCD measurements were performed on ${\mathrm{CD}}_{2}{\mathrm{Cl}}_{2}$ solutions with a Jasco FVS6000 VCD apparatus. In the mid‐IR region ${\mathrm{BaF}}_{2}$ , cells with path length 200 μm or 500 μm were used. A liquid N_2_‐cooled MCT detector was mounted on the instrument, the concentration of the solutions was approx. 0.04 M. For each sample, 6000 scans were carried out for the VCD spectra and 16 scans for absorption ones. The solvent spectra were taken under the same conditions. The latter were subtracted from the spectra in solution for both IR absorption and VCD.

Unless otherwise specified, calculations were performed with the gaussian16 suite of quantum chemistry programs.^[57]^ In the calculations, the combination of the B3PW91^[58]^ functional and TZVP basis sets was employed.^[59]^ The initial geometries for each complex were built starting from the crystallographic structure reported in Refs. [14, 35]. Geometry optimizations were performed with tight convergence criteria (i. e. 1×10^−5^ hartree/bohr and 4×10^−5^ bohr on RMS forces and displacements, respectively, with thresholds for the maximum values being 1.5 times larger) and the minima were confirmed by Hessian evaluations. The harmonic energies and intensities were obtained using the analytical second derivatives of the energy and first derivatives of the properties of interest. Anharmonic calculations using a reduced‐dimensionality (RD) scheme within the second‐order vibrational perturbation theory (VPT2)[^60^, ^61^] were performed with a development version of the gaussian suite of programs.^[62]^ Divergent terms are removed from the perturbative expressions (therefore leading to the so‐called IDVPT2 level).^[63]^

MCD 𝔅 terms were calculated with the origin‐independent SOS approach^[45]^ including the lowest 40 excited states as implemented in the elements library.^[64]^ MCD spectra were then simulated employing the following equation:

Being $\frac{\Delta \epsilon (\omega )}{B_{z}}$ the MCD spectrum, $B_{z}$ the magnetic field oriented along the light beam, $N_{A}$ the Avogadro's number, ω the circular frequency of the radiation, $\epsilon_{0}$ the vacuum permittivity, ℏ the reduced Planck constant, $c_{0}$ the speed of light, $g_{j}\left(\omega \right)$ a Gaussian distribution function centered at the frequency of the j ‐th transition, and 𝔅_*j*_ the 𝔅 term of the j ‐th transition.^[65]^

Vibronic calculations were performed by employing both the sum‐over‐states (or time‐independent, TI) and the time‐dependend (TD) approaches obtaining comparable results.[^66^, ^67^] A Cartesian description was used and the gaussian16 standard parameters were employed. Following standard protocols, the first five modes below 50 cm^−1^ were excluded from the simulations. In TI approach the progression, estimated by comparing the total intensity reached with the transitions actually included with the total intensity known analytically by application of sum rules and closure relations, was satisfactory of 94.8 %.

## Appendix: VCD With Low‐Lying Electronic States (LLESs)

In Nafie's description, under the assumption that vibrational levels are not much different between the lower excited states and the ground states, the contribution of the interaction between the LLES and the vibrational fundamental transition within the ground electronic state at frequency $\omega_{a}$ may be seen as a perturbation onto the $|\psi_{g}\rangle$ ground electronic state of the close‐by excited electronic state $|\psi_{e}\rangle$ and thus treated with a frequency dependent correction terms to be added to the electronic contribution to the atomic polar ($E_{r}^{A}$ ) and axial tensors ($I^{A}$ ). Following the notation presented in Nafie's paper, the corrections to the APTs and AATs are:
(6)

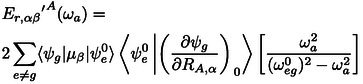



(7)

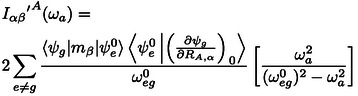




where $R_{A,\alpha }$ is the displacement of atom *A* along the α ‐axis. To be added to the unperturbed terms, which can be readily calculated with magnetic field perturbation (MFP) theory (available in several quantum chemical packages).

For practical applications, the occurence of singularities can be considerably reduced by adding damping factors as done in Ref. [49]. One should substitute in Eqs. (6) and (7) $\omega_{a}$ with $\omega_{a}+i\gamma_{a}$ and $\omega_{eg}^{0}$ with $\omega_{eg}^{0}+i\Gamma_{e}$ , with $\gamma_{a}$ and $\Gamma_{e}$ being damping terms for vibrational and electronic frequencies respectively.

## Supporting Information

The authors report additional spectroscopic and chiroptical data and results of calculations as specified in the text.

## Conflict of Interests

The authors declare no conflict of interest.

1

## Supporting information

As a service to our authors and readers, this journal provides supporting information supplied by the authors. Such materials are peer reviewed and may be re‐organized for online delivery, but are not copy‐edited or typeset. Technical support issues arising from supporting information (other than missing files) should be addressed to the authors.

Supporting Information

## Data Availability

The data that support the findings of this study are available from the corresponding author upon reasonable request.
