# Supplementary material for: Natural and Magnetic Circular Dichroism From the Infrared to the UV of a Hetero[4]helicene Radical Cation
Source: Chem Asian J. 2025 Mar 12;20(10):e202401752. doi: 10.1002/asia.202401752 (PMC12128020; doi:10.1002/asia.202401752)
Supplement: Supplementary file 1 — Supporting Information [file ASIA-20-e202401752-s001.pdf]

Chemistry - An Asian Journal

Supporting Information

Natural and Magnetic Circular Dichroism From the Infrared to the UV of a Hetero[4]helicene Radical Cation

Marco Fusè, Michela Lupi, Ewa Machalska, Giuseppe Mazzeo, Sergio Abbate,* Julien Bloino, Caterina Viglianisi, Stefano Menichetti,* and Giovanna Longhi*

Supporting Information: Natural and Magnetic Circular Dichroism from the Infrared to the UV of a Hetero[4]Helicene Radical Cation

Marco Fusè,† Michela Lupi,‡ Ewa Machalska,† Giuseppe Mazzeo,† Sergio Abbate,†,§ Julien Bloino,¶ Caterina Viglianisi,‡ Stefano Menichetti,*,‡ and Giovanna Longhi*,†,‖

†*Dipartimento di Medicina Molecolare e Traslazionale (DMMT), Università di Brescia, Viale Europa 11, 25123 Brescia, Italy*

‡*Dipartimento di Chimica, Università di Firenze, Via della Lastruccia 13, 50019 Sesto Fiorentino, Italy*

¶*Scuola Normale Superiore, Piazza dei Cavalieri 8, 56125, Pisa, Italy*

§*Istituto Nazionale di Ottica (INO), CNR, Research Unit Brescia, c/o CSMT via Branze 45, 25123 Brescia, Italy*

‖*Istituto Nazionale di Ottica (INO), CNR, Research Unit Brescia, c/o CSMT via Branze, 45 – 25123 Brescia, Italy*

E-mail: stefano.menichetti@unifi.it; giovanna.longhi@unibs.it

S1 Additional Experimental Spectra

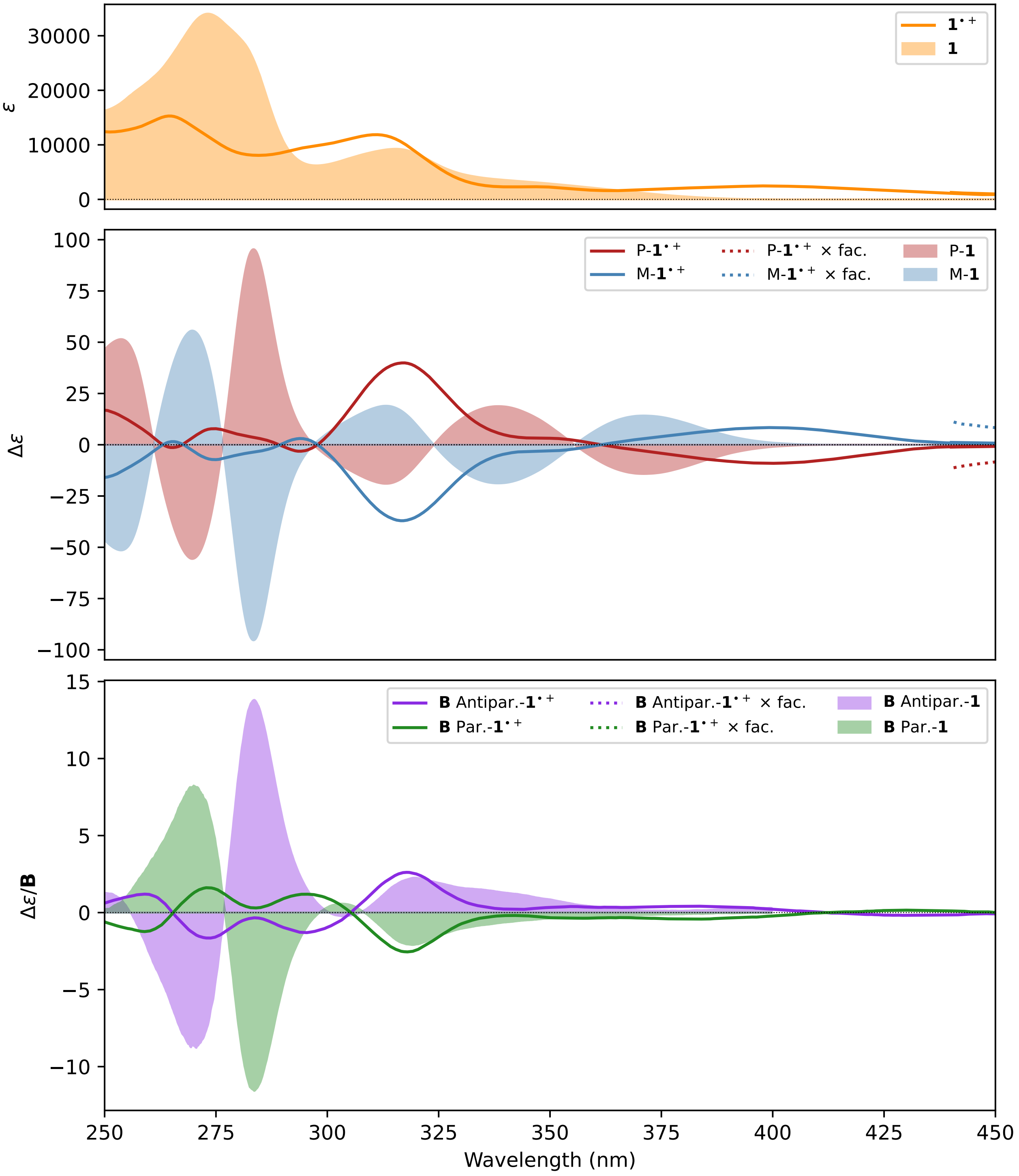

Figure S1: UV-Vis (top panel), ECD (middle panel) and MCD (bottom panel) experimental spectra of **1** and $\mathbf{1}^{\bullet+}$. Measurements were performed on a CH_2Cl_2 solution in 250–450 nm range. **1** spectra are reported as shaded areas and $\mathbf{1}^{\bullet+}$ spectra as lines.

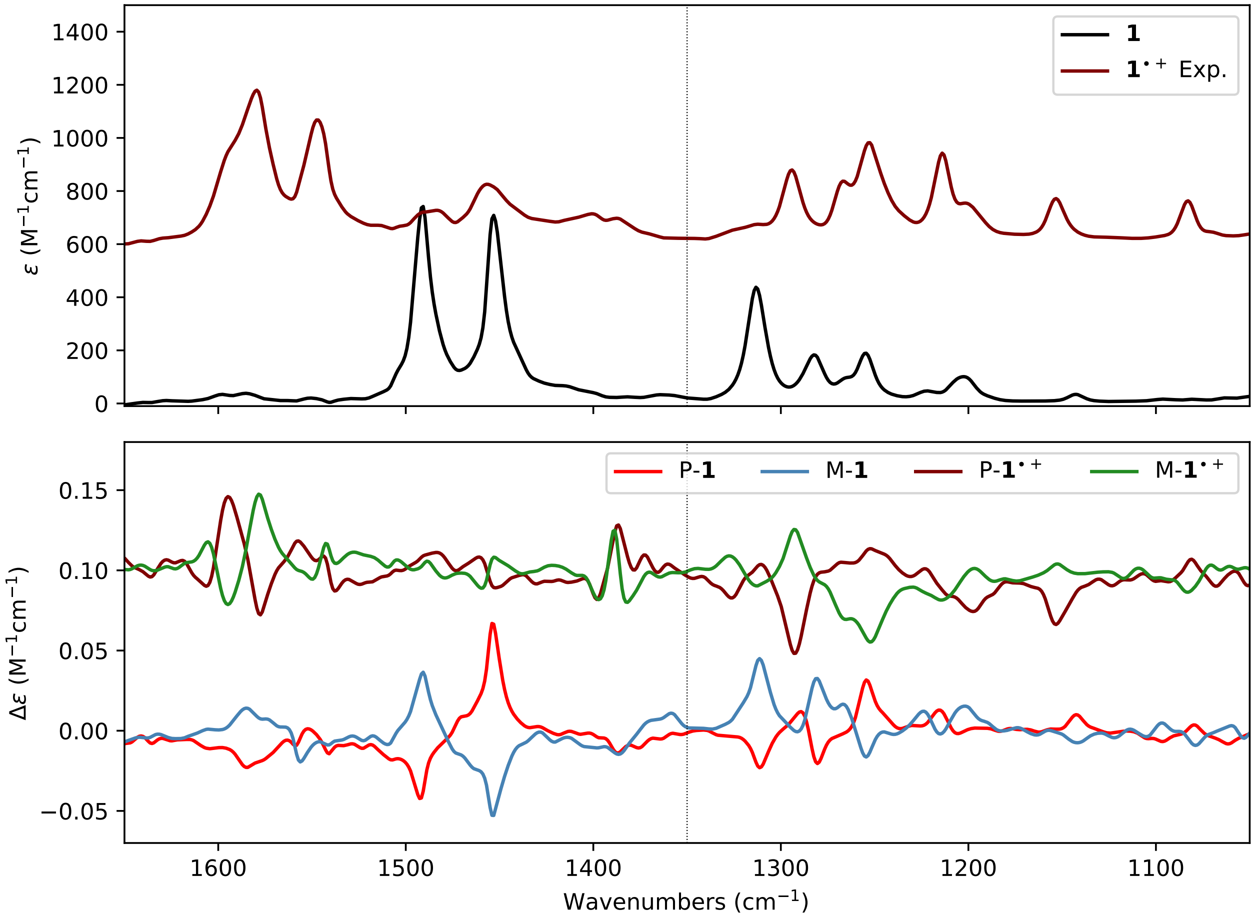

Figure S2: IR absorption and VCD experimental spectra. CD_2Cl_2 solutions with cells of path length of 200 μm and 500 μm were employed for the two species respectively.

S2 Additional Computational data

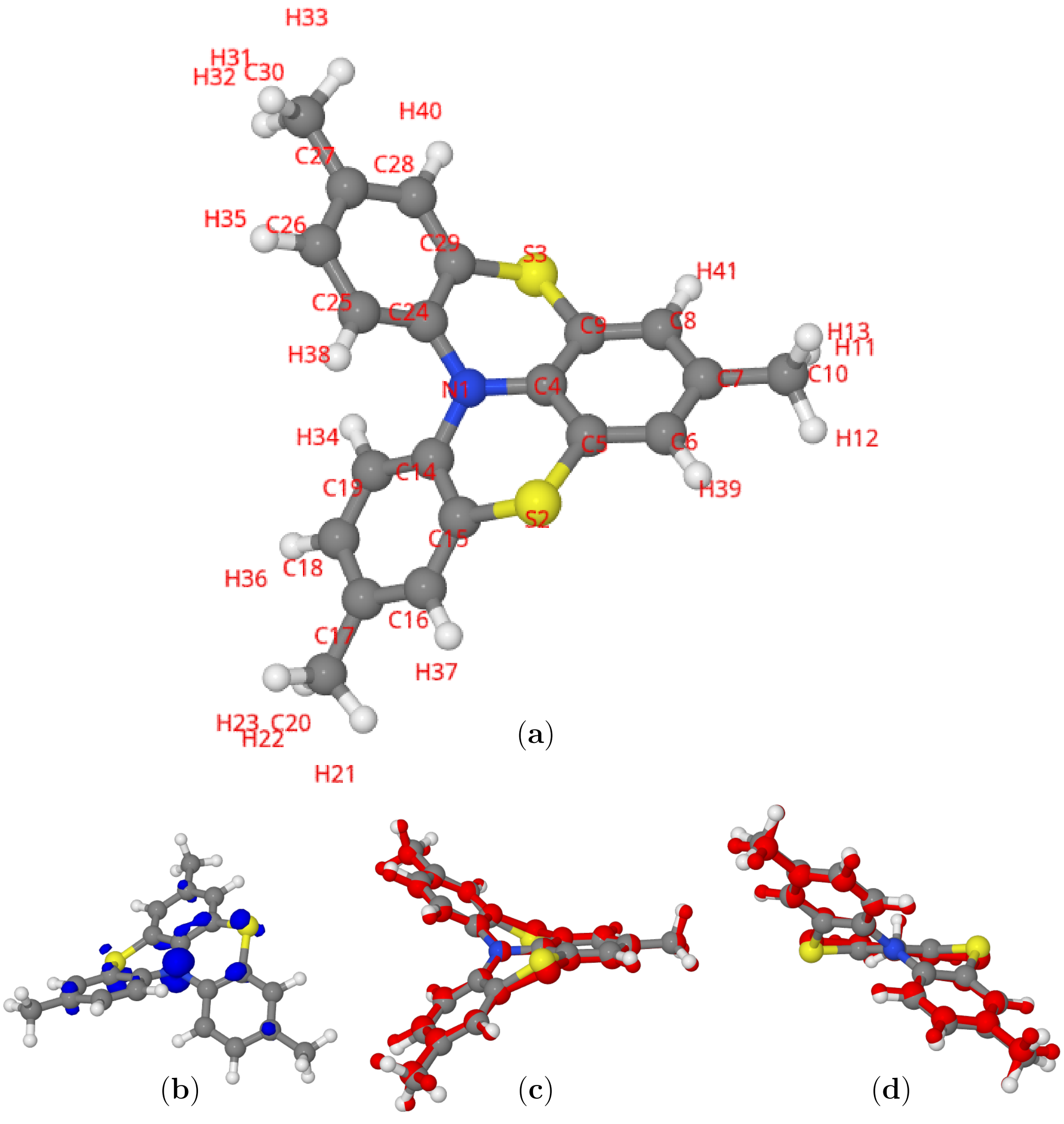

Figure S3: (**a**)Atom numbering in the structures used across the article; (**b**) Calculated isodensity spin surfaces (blue) (iso value of 0.07 e Bohr^{-3}); (**c**) and (**d**) two views of **1** and $\mathbf{1}^{\bullet+}$ (in red) superimposed calculated structures.

Table S1: Wavelength (nm), C_2 species symmetry, electric (EDTM) and magnetic (MDTM) dipole transition moments (scaled cgs units), dipole and rotational strength (scaled cgs units) and $\mathfrak{B}$ (atomic units) of the first 10 electronic transitions of **1** and $\mathbf{1}^{\bullet+}$. The C_2 axis lays on the y Cartesian axis.

	Wavel.	Sym.	EDTM x	EDTM y	EDTM z	MDTM x	MDTM y	MDTM z	DS	RS	$\mathfrak{B}$
Neutral species **1**											
1	356.51	B	1.26667e-18	-2.58768e-20	-5.29655e-19	-6.28100e-21	-2.10581e-22	1.15712e-20	1.88566e-36	-1.40793e-38	-1.36776e+00
2	341.80	A	5.24379e-20	7.68878e-19	-5.14082e-20	-3.71844e-23	5.82685e-21	-8.37450e-23	5.96565e-37	4.48249e-39	-6.75564e+00
3	336.77	B	7.16048e-19	-2.56394e-20	-9.25710e-19	2.41594e-21	-1.81516e-22	-9.26353e-21	1.37032e-36	1.03099e-38	-5.71102e+00
4	310.28	B	-2.90188e-18	-3.10209e-20	-4.74300e-19	2.76985e-21	-3.53713e-23	-5.59709e-21	8.64680e-36	-5.38197e-39	-1.14131e+01
5	299.34	A	7.38272e-21	1.19452e-18	-1.13147e-20	-1.42663e-23	-5.38212e-23	-2.41522e-23	1.42706e-36	-6.41225e-41	2.05256e+01
6	279.11	A	2.29897e-19	-2.45758e-18	-7.56204e-21	6.63135e-22	5.04904e-21	-1.06113e-21	6.09261e-36	-1.22479e-38	-4.51015e+02
7	278.22	B	-2.86601e-18	-1.48173e-19	1.09038e-19	-7.80936e-21	3.10781e-22	1.26180e-20	8.24788e-36	2.37115e-38	4.53918e+02
8	269.47	A	2.93962e-20	2.92229e-18	-9.57118e-21	6.30148e-23	-1.61441e-21	-7.29967e-23	8.54076e-36	-4.71523e-39	2.75810e+01
9	261.19		-1.53593e-19	3.48573e-19	-1.68433e-20	-5.70313e-22	-1.52882e-21	2.27330e-21	1.45378e-37	-4.83598e-40	3.30620e+00
10	259.60		4.58577e-19	1.45916e-19	6.11879e-20	1.71160e-21	-2.82861e-22	-9.20906e-21	2.35329e-37	1.80144e-40	1.79162e+00
Radical-cation species $\mathbf{1}^{\bullet+}$											
1	1151.89	B	-2.95252e-18	-1.35852e-20	-7.18343e-19	2.96421e-22	2.19148e-23	-1.74298e-21	9.23356e-36	3.76574e-40	1.00750e+01
2	719.63	A	7.26951e-21	-1.58385e-18	4.99494e-21	3.75014e-24	1.67658e-21	-2.16409e-23	2.50866e-36	-2.65554e-39	9.72298e+00
3	574.68	A	-3.54129e-20	3.10314e-18	2.39707e-20	8.02070e-23	7.97459e-22	1.20619e-23	9.63130e-36	2.47207e-39	-4.78684e+01
4	537.52	B	-3.19885e-18	-2.23668e-20	6.42176e-19	4.08161e-22	-9.22451e-24	-2.19039e-22	1.06455e-35	-1.44610e-39	3.08477e+01
5	472.04	B	6.21973e-21	1.05388e-20	7.09348e-20	-2.11306e-22	1.52188e-23	2.52855e-22	5.18150e-39	1.67823e-41	-1.21842e-01
6	392.77	A	-3.08786e-21	-2.25948e-19	1.84503e-20	8.10287e-23	-5.46398e-22	1.15149e-23	5.14025e-38	1.23420e-40	5.63940e-01
7	383.67	A	5.33422e-22	-9.11830e-19	-1.68544e-21	2.74084e-23	-2.15676e-21	-6.28721e-23	8.31436e-37	1.96672e-39	1.79323e+00
8	374.74	B	1.56819e-18	-1.35098e-21	-2.13456e-19	-3.19268e-21	8.17767e-24	7.64648e-21	2.50480e-36	-6.63894e-39	-6.80165e+00
9	349.67	A	4.05331e-21	-6.65201e-20	-6.51801e-21	-1.11208e-23	-1.72169e-21	1.83894e-23	4.48384e-39	1.14362e-40	-2.59503e-01
10	333.91		1.36861e-20	5.55165e-20	8.26346e-21	7.15535e-23	-1.78756e-21	-9.95124e-23	3.33768e-39	-9.90819e-41	6.15927e-01

Table S2: NM 100: electric and magnetic dipole transition moments (cgs), dipole and rotational strength (cgs) of **1** and $\mathbf{1}^{\bullet+}$. The C_2 axis lays on the y Cartesian axis.

NM: 100								
Neutral (ν = 1660.6 cm^{-1})								
	$\boldsymbol{\mu} \cdot 10^{20}$			$\mathbf{m} \cdot 10^{24}$			DS $\cdot 10^{40}$	RS $\cdot 10^{44}$
	x	y	z	x	y	z		
tot	0.05	-0.30	-0.04	-0.01	4.04	0.02	0.09	-1.21
ele	0.13	-3.92	-0.21	-0.19	3.77	-0.07	15.43	-14.78
nuc	-0.07	3.62	0.16	0.18	0.27	0.09	13.14	0.99
Radical cation (ν = 1653.4 cm^{-1})								
	x	y	z	x	y	z		
tot	0.00	4.98	-0.06	0.01	-5.82	-0.01	24.77	-28.98
ele	0.10	9.31	-0.35	-0.40	-7.41	-0.18	86.84	-69.01
nuc	-0.10	-4.33	0.30	0.41	1.59	0.17	18.89	-6.88

Table S3: NM 99: electric and magnetic dipole transition moments (cgs), dipole and rotational strength (cgs) of **1** and $\mathbf{1}^{\bullet+}$. The C_2 axis lays on the y Cartesian axis.

NM: 99								
Neutral (ν = 1649.6 cm^{-1})								
	$\boldsymbol{\mu} \cdot 10^{20}$			$\mathbf{m} \cdot 10^{24}$			DS $\cdot 10^{40}$	RS $\cdot 10^{44}$
	x	y	z	x	y	z		
tot	4.74	0.00	1.25	-8.97	-0.09	20.68	24.05	-16.80
ele	10.79	0.00	2.68	-9.03	-0.10	22.14	123.59	-38.05
nuc	-6.05	-0.00	-1.44	0.06	0.01	-1.46	38.62	1.74
Radical cation (ν = 1640.6 cm^{-1})								
	x	y	z	x	y	z		
tot	-21.05	-0.11	4.02	-3.13	0.02	15.82	459.11	129.35
ele	-12.19	-0.05	6.94	-4.05	0.01	18.74	196.75	179.41
nuc	-8.86	-0.06	-2.93	0.92	0.01	-2.92	87.03	0.39

Table S4: NM 98: electric and magnetic dipole transition moments (cgs), dipole and rotational strength (cgs) of **1** and **1**$^{\bullet+}$. The C_2 axis lays on the y Cartesian axis.

NM: 98								
Neutral (ν = 1633.3 cm^{-1})								
	$\boldsymbol{\mu} \cdot 10^{20}$			$\mathbf{m} \cdot 10^{24}$			DS $\cdot 10^{40}$	RS $\cdot 10^{44}$
	x	y	z	x	y	z		
tot	0.01	-6.08	0.08	0.13	6.90	-0.10	36.94	-41.94
ele	-0.16	-0.08	0.39	0.53	7.24	0.12	0.18	-0.64
nuc	0.17	-5.99	-0.31	-0.40	-0.34	-0.22	36.06	2.02
Radical cation (ν = 1626.2 cm^{-1})								
	x	y	z	x	y	z		
tot	0.28	-28.88	-0.11	-0.13	5.44	-0.13	834.00	-157.14
ele	0.52	-21.58	-0.87	-1.23	6.34	-0.56	466.59	-137.01
nuc	-0.24	-7.30	0.76	1.09	-0.90	0.43	53.93	6.66

Table S5: NM 93: electric and magnetic dipole transition moments (cgs), dipole and rotational strength (cgs) of **1** and **1**$^{\bullet+}$. The C_2 axis lays on the y Cartesian axis.

NM: 93								
Neutral (ν = 1530.9 cm^{-1})								
	$\boldsymbol{\mu} \cdot 10^{20}$			$\mathbf{m} \cdot 10^{24}$			DS $\cdot 10^{40}$	RS $\cdot 10^{44}$
	x	y	z	x	y	z		
tot	27.72	-0.02	-4.56	-5.06	-0.08	7.13	789.19	-172.76
ele	11.05	-0.09	4.70	-4.64	-0.02	3.48	144.18	-34.93
nuc	16.67	0.07	-9.26	-0.42	-0.06	3.65	363.78	-40.72
Radical cation (ν = 1497.6 cm^{-1})								
	x	y	z	x	y	z		
tot	-9.73	-0.15	-5.64	5.41	-0.10	-20.09	126.56	60.71
ele	-17.30	-0.34	7.14	7.17	-0.17	-21.27	350.41	-275.92
nuc	7.57	0.19	-12.78	-1.76	0.07	1.18	220.65	-28.44

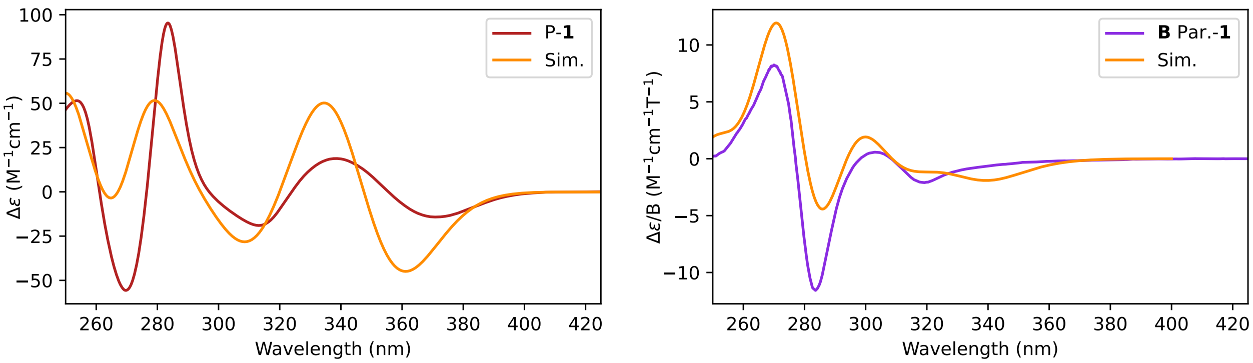

Figure S4: Comparison of experimental and simulated ECD and MCD spectra of **1** (*P* enantiomer and racemic form respectively).

Table S6: NM 90: electric and magnetic dipole transition moments (cgs), dipole and rotational strength (cgs) of **1** and $\mathbf{1}^{\bullet+}$. The C_2 axis lays on the y Cartesian axis.

NM: 90<br>Neutral ($\nu = 1489.7$ cm^{-1})								
	$\boldsymbol{\mu} \cdot 10^{20}$			$\mathbf{m} \cdot 10^{24}$			DS $\cdot 10^{40}$	RS $\cdot 10^{44}$
	x	y	z	x	y	z		
tot	-0.20	25.64	0.11	2.00	2.38	-2.45	657.37	60.37
ele	2.26	0.21	-1.30	-2.61	9.51	1.60	6.84	-5.93
nuc	-2.46	25.42	1.41	4.61	-7.13	-4.05	654.40	-198.19
NM: 86<br>Radical cation ($\nu = 1449.6$ cm^{-1})								
	x	y	z	x	y	z		
tot	0.04	-2.98	-0.20	-0.30	5.00	-0.05	8.93	-14.92
ele	-0.05	17.66	-0.08	-0.14	6.34	-0.08	311.96	111.98
nuc	0.09	-20.64	-0.12	-0.16	-1.34	0.04	426.18	27.61

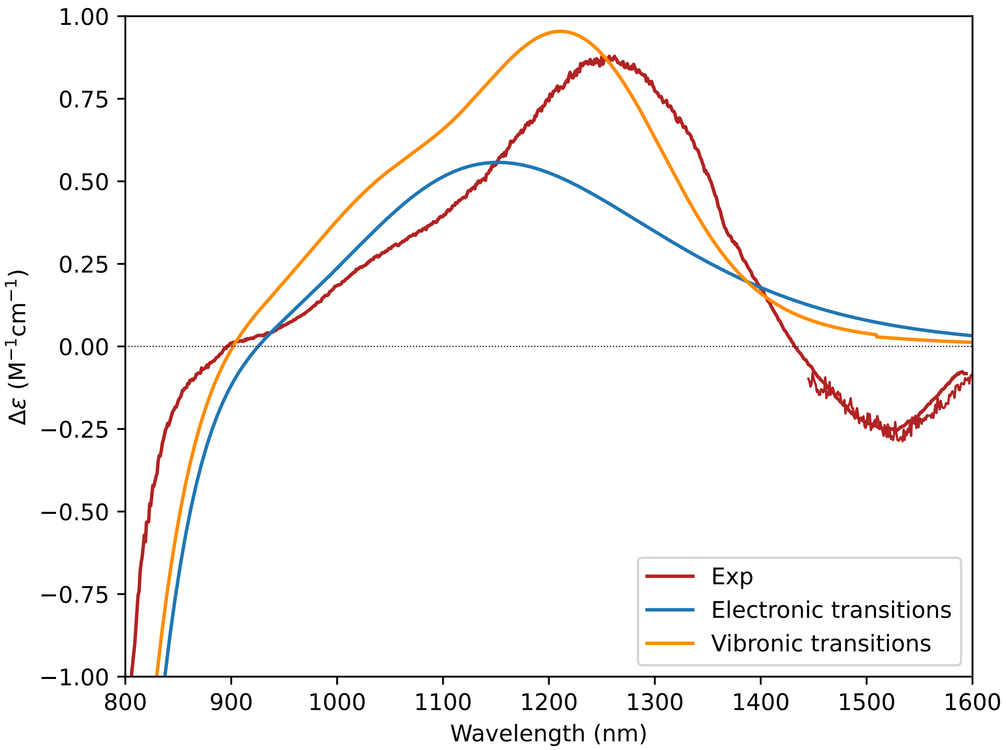

Figure S5: Comparison between the simulated spectra of $\mathbf{1}^{\bullet+}$. The first transition were treated with and without vibronic contribution at AH—FCHT level. HWWM of 1200 cm^{-1} was used for pure electronic transition and a HWHM of 135 cm^{-1} for modelling the vibronic transition. Notice that the trace affected by higher experimental error between 1400–1600 nm was obtained with a different detector.

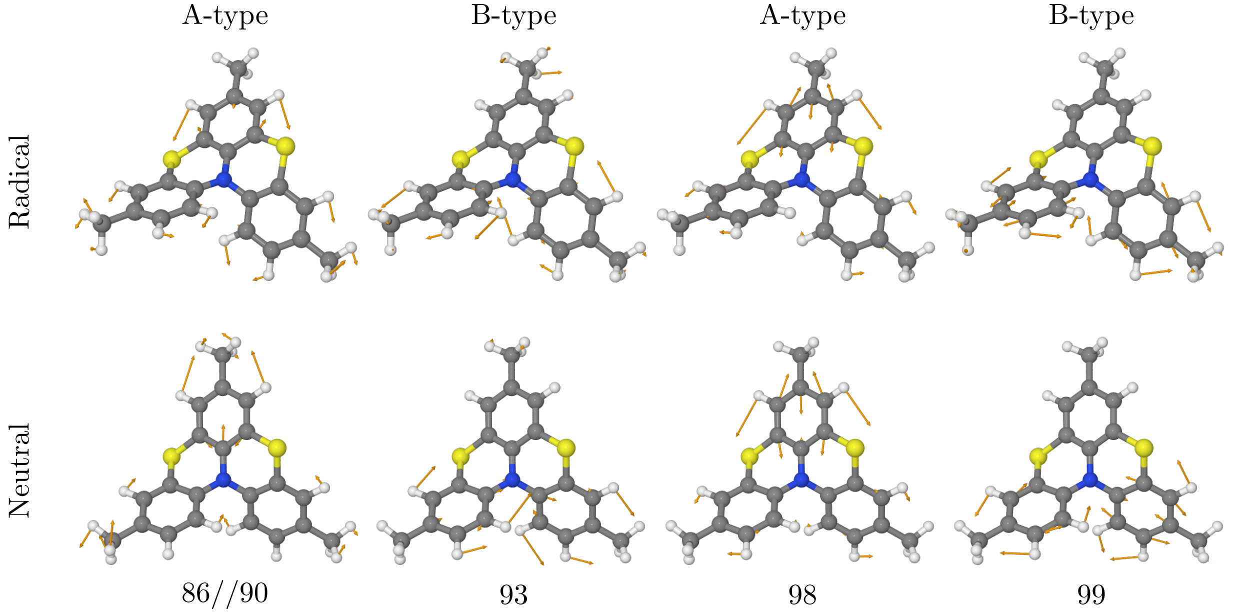

Figure S6: Graphical representation of four selected normal modes of the neutral and radical forms.

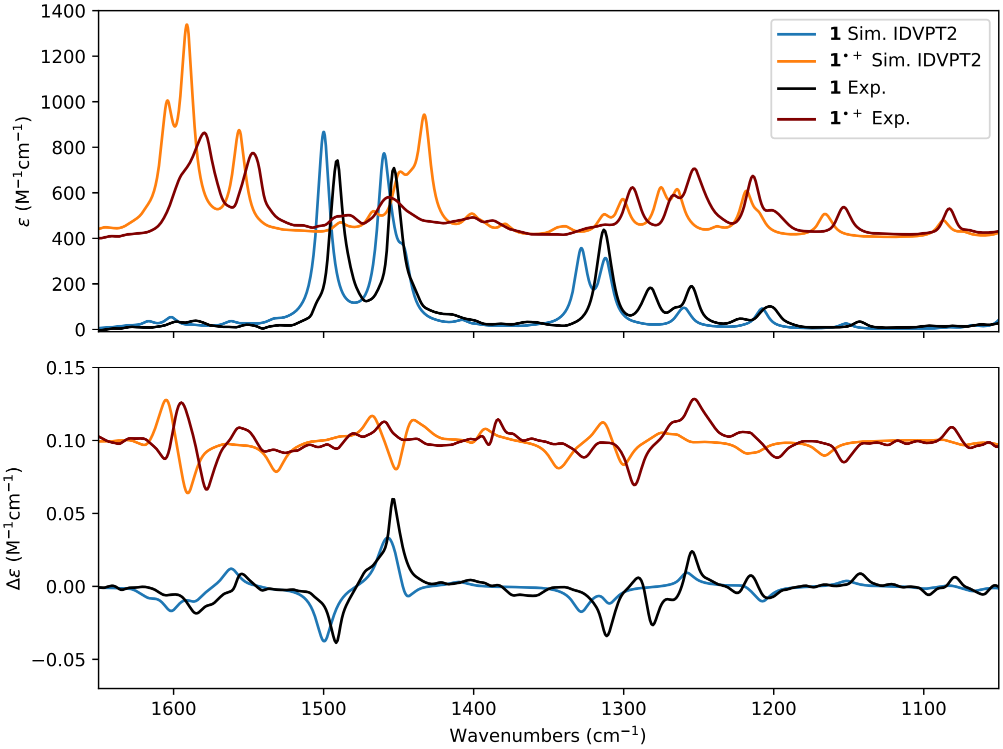

Figure S7: Comparison of experimental and simulated absorption and VCD spectra of **1** (Neu.) and $\mathbf{1}^{\bullet+}$ (Rad.) *P* enantiomer. Anharmonic calculations (IDVPT2)[S1] were performed at the B3PW91/TZVP level of theory. The spectral band-shape was obtained by applying Lorentzian distribution functions with 5 cm^{-1} half-width at half-maximum.

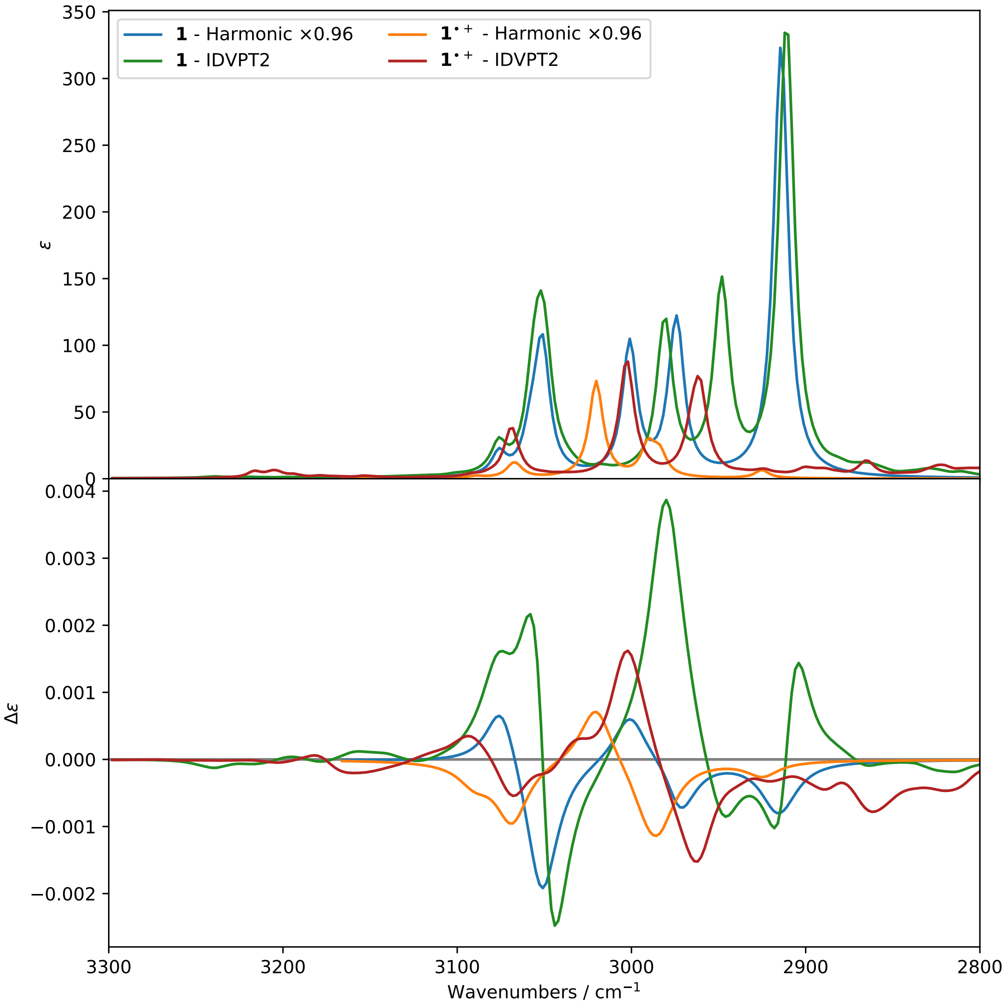

Figure S8: Simulated VA and VCD spectra of **1** and **1**$^{\bullet+}$ in the CH-stretching region. The spectral band-shape was obtained was obtained by applying Lorentzian distribution functions with 12 cm^{-1} half-width at half-maximum. A scaling factor of 0.96 was applied to the harmonic frequencies. It is worth noting that VA spectrum of **1**$^{\bullet+}$ is predicted to be one order of magnitude less intense than the finger print region (see Figure S7). Thus, making it difficult to compensate the CD_2Cl_2 peaks laying in the same region for **1**$^{\bullet+}$. Interestingly, going from the neutral to the radical form, the intensity of the most intense peak associated with CH_3 stretching sensibly decreases, being only one fourth of the one predicted for the neutral form, as indeed experimentally observed.

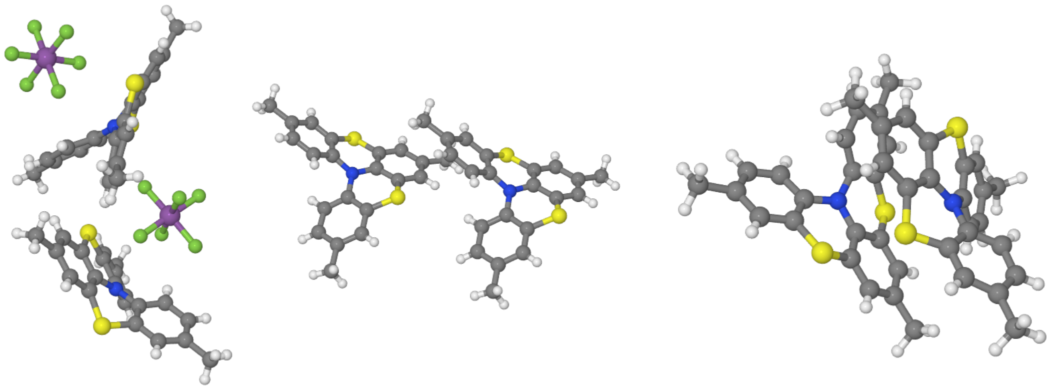

a **b** **c**

Figure S9: Graphical representation of the three extracted dimers from the crystallographic structures (see main text). **a** and **b** from ref. S2, **c** adapted from ref. S3.

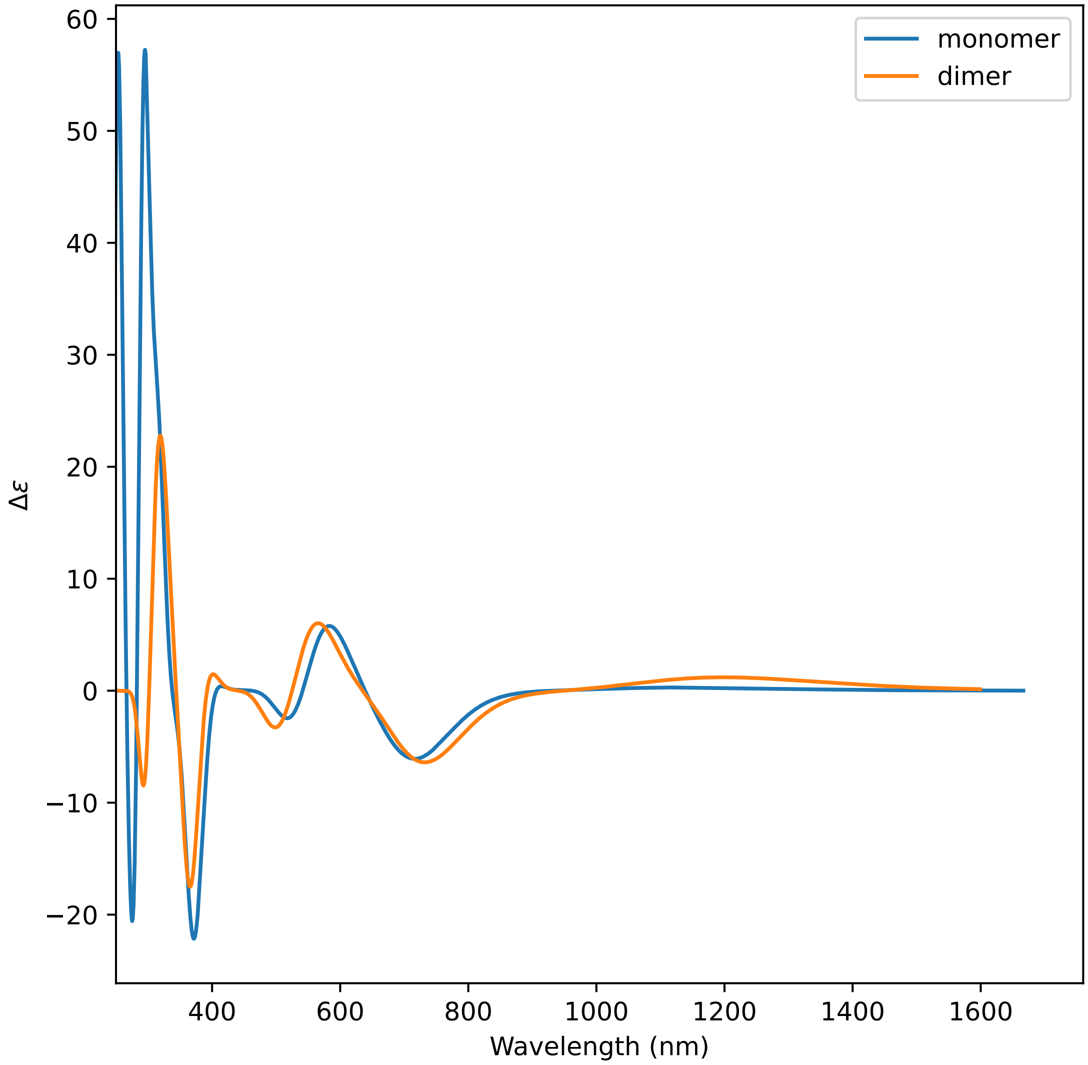

Figure S10: Comparison of the ECD spectra computed with monomer structure and a dimer structure (first structure on the left **a** of Figure S9 was employed) of $\mathbf{1}^{\bullet+}$. The counter ions were included in the simulation of the dimer and the triplet state was simulated.

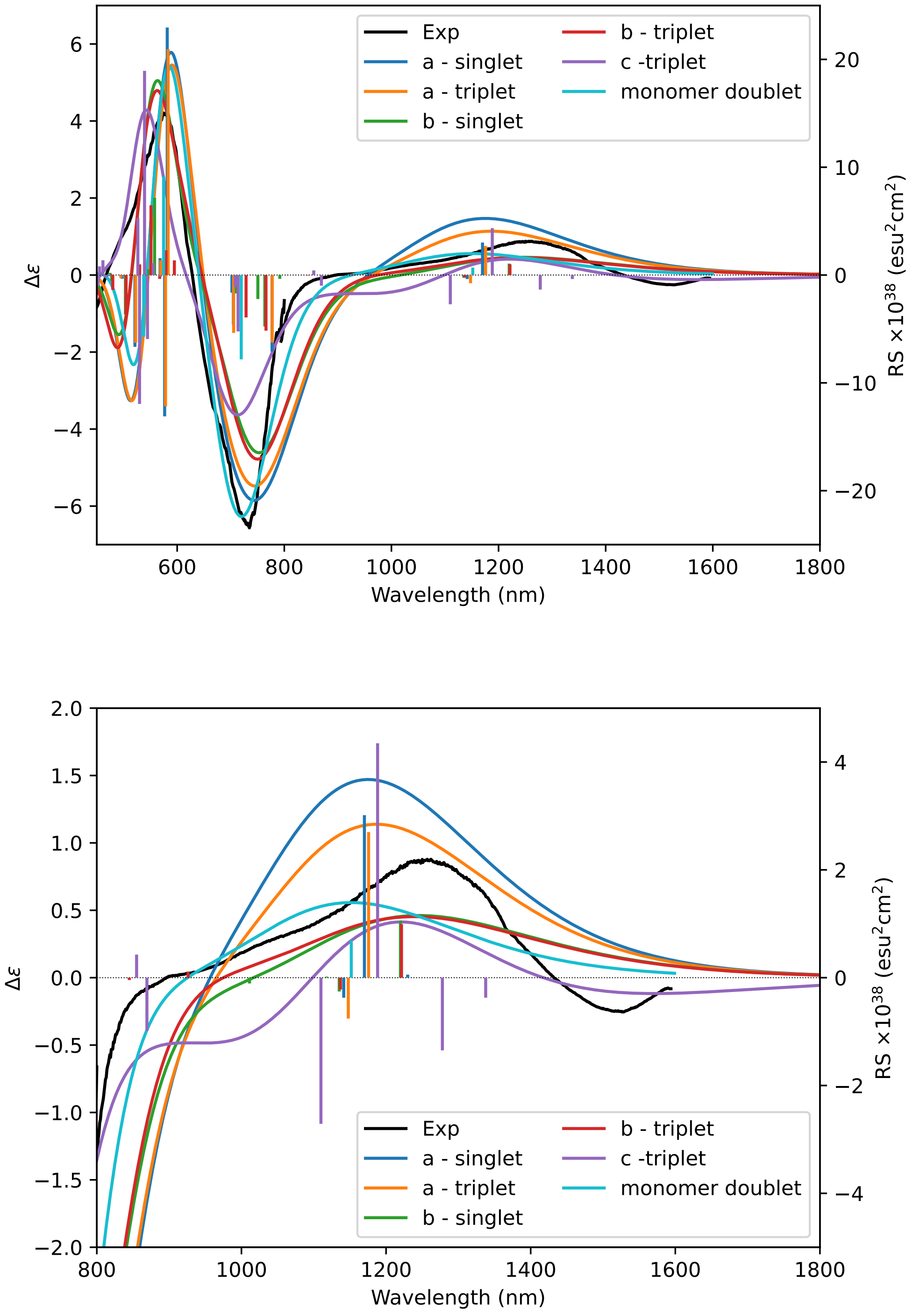

Figure S11: Comparison of the ECD spectra computed with monomer structure and a dimer structure (first structure on the left **a** of Figure S9 was employed) of $\mathbf{1}^{\bullet +}$. The counter ions were excluded in the simulation of the dimer and the singlet and triplet states were simulated.

S3 Cartesian Coordinates

1

```
41
geom - modified
7        0.00688963       0.00316959      -0.23676767
16      -1.40468180      -2.38834194       0.91451500
16       1.41176705       2.30640071       1.09009344
6       -0.00399982      -0.04291188       1.17161364
6       -0.58821425      -1.11342226       1.84766399
6       -0.56868272      -1.17309633       3.23602790
6       -0.02035302      -0.13819260       3.98950059
6        0.54044078       0.94281259       3.31403490
6        0.57428389       0.97804507       1.92520677
6       -0.05995517      -0.17287022       5.49133466
1        0.78256496       0.36995063       5.92392816
1       -0.03044108      -1.19779095       5.86595466
1       -0.97731000       0.28874342       5.87043184
6        0.20340334      -1.18781753      -0.96640201
6       -0.35411296      -2.38750801      -0.52156966
6       -0.12757679      -3.57202417      -1.21384860
6        0.62112159      -3.59053571      -2.38681829
6        1.17813576      -2.38729690      -2.82220330
6        0.99164025      -1.21016093      -2.11672292
6        0.81254288      -4.86256534      -3.16297171
1        0.70181845      -5.74027727      -2.52366828
1        1.80236792      -4.90205662      -3.62276333
1        0.07543310      -4.94786189      -3.96805792
6       -0.17355125       1.24088742      -0.88884825
6       -0.94510920       1.34294131      -2.04610286
6       -1.11598935       2.56480162      -2.67535200
6       -0.55966123       3.73374207      -2.15402571
6        0.17192411       3.63424464      -0.97436277
6        0.38295248       2.40559382      -0.35821450
6       -0.73409111       5.05499671      -2.84754638
1       -1.71737605       5.13042243      -3.31678251
1        0.01428827       5.18837368      -3.63551534
1       -0.62753781       5.88827677      -2.15067753
1        1.45306439      -0.29087279      -2.45708299
1       -1.71332929       2.61291464      -3.58001146
1        1.78842416      -2.37306328      -3.71936021
1       -0.55491643      -4.49279522      -0.83028380
1       -1.40563756       0.45037857      -2.45246320
1       -1.00099002      -2.03556492       3.73181316
1        0.59776817       4.52553039      -0.52511164
1        0.97408640       1.76564320       3.87218005
```

$1^{\bullet+}$

41
Enegy: -1661.27026533

N	0.010923	0.000349	-0.150016
S	-1.141059	-2.557000	1.071070
S	1.157749	2.470402	1.241824
C	0.003832	-0.044190	1.244417
C	-0.448618	-1.188133	1.930785
C	-0.449317	-1.221909	3.316872
C	-0.005907	-0.135590	4.068709
C	0.446665	0.995208	3.391765
C	0.452349	1.052450	2.006511
C	-0.040381	-0.172181	5.565196
H	0.742311	0.451443	5.998375
H	-1.001238	0.203126	5.931401
H	0.078972	-1.188679	5.941847
C	0.202341	-1.175668	-0.901163
C	-0.281476	-2.412690	-0.452181
C	-0.115777	-3.553053	-1.235388
C	0.553366	-3.503067	-2.449873
C	1.084919	-2.268766	-2.860625
C	0.916061	-1.130554	-2.108085
C	0.736537	-4.728554	-3.289149
H	0.328267	-5.615075	-2.804821
H	1.795516	-4.906855	-3.492475
H	0.239192	-4.611059	-4.256100
C	-0.172941	1.222767	-0.825437
C	-0.877465	1.256934	-2.038016
C	-1.038538	2.441663	-2.716931
C	-0.507796	3.645698	-2.223618
C	0.152141	3.615926	-1.003445
C	0.310096	2.427027	-0.294267
C	-0.682176	4.923057	-2.983721
H	-1.738983	5.115299	-3.185552
H	-0.278245	5.775989	-2.439174
H	-0.175284	4.867708	-3.951279
H	1.350592	-0.194365	-2.433928
H	-1.602159	2.446437	-3.643064
H	1.655493	-2.212838	-3.780804
H	-0.500314	-4.499328	-0.871346
H	-1.311107	0.344576	-2.426650
H	-0.797360	-2.116908	3.820163
H	0.535604	4.535906	-0.576343
H	0.797800	1.852958	3.954348

References

(S1) Yang, Q.; Mendolicchio, M.; Barone, V.; Bloino, J. Accuracy and Reliability in the Simulation of Vibrational Spectra: A Comprehensive Benchmark of Energies and Intensities Issuing From Generalized Vibrational Perturbation Theory to Second Order (GVPT2). *Front. Astron. Space Sci.* **2021**, *8*, 665232.

(S2) Lamanna, G.; Faggi, C.; Gasparrini, F.; Ciogli, A.; Villani, C.; Stephens, P. J.; Devlin, F. J.; Menichetti, S. Efficient Thia-Bridged Triarylamine Heterohelicenes: Synthesis, Resolution, and Absolute Configuration Determination. *Chemistry - A European Journal* **2008**, *14*, 5747–5750.

(S3) Gliemann, B. D.; Petrovic, A. G.; Zolnhofer, E. M.; Dral, P. O.; Hampel, F.; Breitenbruch, G.; Schulze, P.; Raghavan, V.; Meyer, K.; Polavarapu, P. L.; Berova, N.; Kivala, M. Configurationally Stable Chiral Dithia-Bridged Hetero[4]helicene Radical Cation: Electronic Structure and Absolute Configuration. *Chemistry – An Asian Journal* **2017**, *12*, 31–35.
